# MRI of a presumptive intracranial histiocytic sarcoma with extracranial extension in a cat

**DOI:** 10.1177/20551169241264134

**Published:** 2024-08-02

**Authors:** Anna Eichrodt, Nico Mauri, Maja Ruetten, Barbara Kaser-Hotz, Susann Dressel-Böhm

**Affiliations:** 1 Vetimage Diagnostik AG, Oberentfelden, Switzerland; 2 Clinic of Diagnostic Imaging, Vetsuisse Faculty, University of Zurich, Zurich, Switzerland; 3 Pathovet AG, Tagelswangen, Switzerland

**Keywords:** Neoplasia, intra-axial, extra-axial, skull, temporal muscle myopathy, leptomeningeal, pachymeningeal

## Abstract

**Case summary:**

A 4-year-old female neutered Scottish Fold shorthair cat was presented for further investigation of circling towards the right. MRI of the brain revealed an extensive, right-sided temporal muscle lesion with associated frontotemporal bone osteolysis, intracranial, extra-axial extension along the calvarial convexity with severe pachy- and leptomeningeal thickening and contrast enhancement, and an intra-axial space-occupying lesion in the right piriform lobe. The regional lymph nodes were moderately enlarged. Cytology of the right parotid lymph node and the temporal muscle was performed and histiocytic sarcoma (HS) was diagnosed. The owners elected euthanasia.

**Relevance and novel information:**

HS of the central nervous system (CNS) is a very rare neoplastic condition in cats. Although a few case reports mention MRI, to our knowledge, the characterisation of MRI features of feline CNS HS have not been investigated in detail. Therefore, the aim of this case report was to describe the MRI characteristics in a feline HS involving not only the CNS, but also the fronto-temporal bone, temporal muscle and the regional lymph nodes. In particular, aggressive neoplastic bone invasion was a novel finding.

## Case description

A 4-year-old female neutered Scottish Fold shorthair cat was presented with a history of reduced general condition in the past week, and acute progressive onset of circling towards the right side. General physical examination was unremarkable. Neurological examination revealed intermittent, non-compulsive circling towards the right, mild ataxic gait and straddled forelimbs when sitting. Proprioception, cranial nerves and dazzle reaction were unremarkable. In addition, there was no pathologic nystagmus. The cat was reduced, but alert and responsive. Complete blood count was within the reference intervals (RIs). Serum biochemistry showed a mild hyperproteinaemia (85 g/l; RI 64–80) and a mild hypercholesterinaemia (8.7 mmol/l; RI 2.6–6.8). Before presentation, the cat was treated with meloxicam (Metacam; Boehringer Ingelheim) and cannabidiol (Calmin 5%; Inuvet) on suspicion of joint pain. The cat also had a history of breed-related osteochondrodysplasia. Furthermore, she had never been outside of Switzerland and was regularly vaccinated and dewormed. Differential diagnoses at that time included a metabolic or inflammatory/infectious disease, an unobserved trauma, a neoplastic process and a vascular anomaly or insult.

MRI of the cat’s brain was performed using a 1.5 Tesla (T) machine (Signa HDxt Excite 1.5T; GE Medical Systems) with a 1.5 T eight-channel Transmit/Receive Knee Array (GE Medical Systems). The following sequences were acquired: dorsal and transverse T2-weighted (T2W) fluid-attenuated inversion recovery (FLAIR); transverse, sagittal and dorsal T2W fast spin echo (FSE); transverse T1-weighted (T1W) FSE; transverse T2* multiecho gradient recalled echo (GRE); transverse diffusion-weighted imaging; and apparent diffusion coefficient mapping. After intravenous administration of a gadolinium-based contrast medium (gadoteric acid, Clariscan 0.5 mmol/ml, 0.2 ml/kg IV; GE Healthcare), transverse fat-saturated T1W FSE and dorsal spoiled gradient recalled echo (SPGR) 3D sequences were obtained.

**Figure 1:**
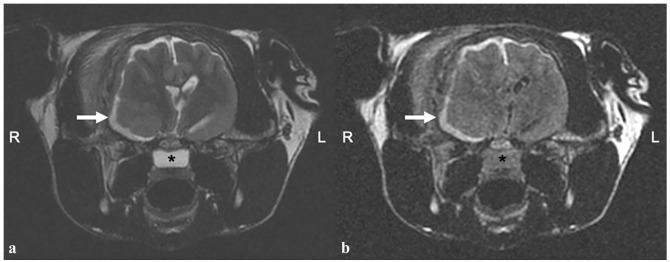
(a) Transverse T2-weighted (T2W), and (b) T2W fluid-attenuated inversion recovery (FLAIR) images of the brain. There is an ill-defined, broad-based space-occupying lesion (arrows) centred at the level of the right piriform lobe that extends along the calvarium. The mass is iso- to faintly hyperintense on T2W and T2W FLAIR images and surrounded by an irregular and focally discontinuous marked T2W and FLAIR hyperintensity that expands along the meninges. The nasopharynx is fluid filled (*)

**Figure 2:**
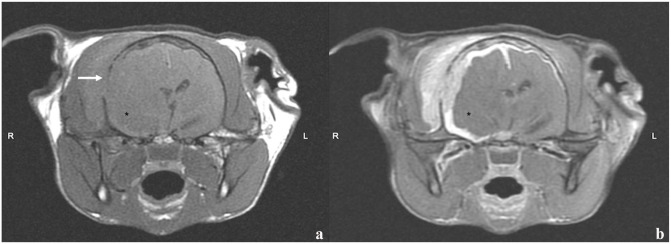
Transverse T1-weighted (T1W) (a) pre- and (b) postcontrast images of the brain. There is diffuse cerebral grey matter swelling along the right cerebral convexity and severe regional to diffuse pachy- and leptomeningeal thickening with severe, mildly heterogeneous, contrast enhancement. The intracranial lesion extends extracranially into a right temporal muscle lesion through an osteolytic defect in the right temporal bone (arrow). The poorly marginated space-occupying lesion (*) at the level of the piriform lobe is T1W iso- to mildly hypointense and not contrast-enhancing

**Figure 3:**
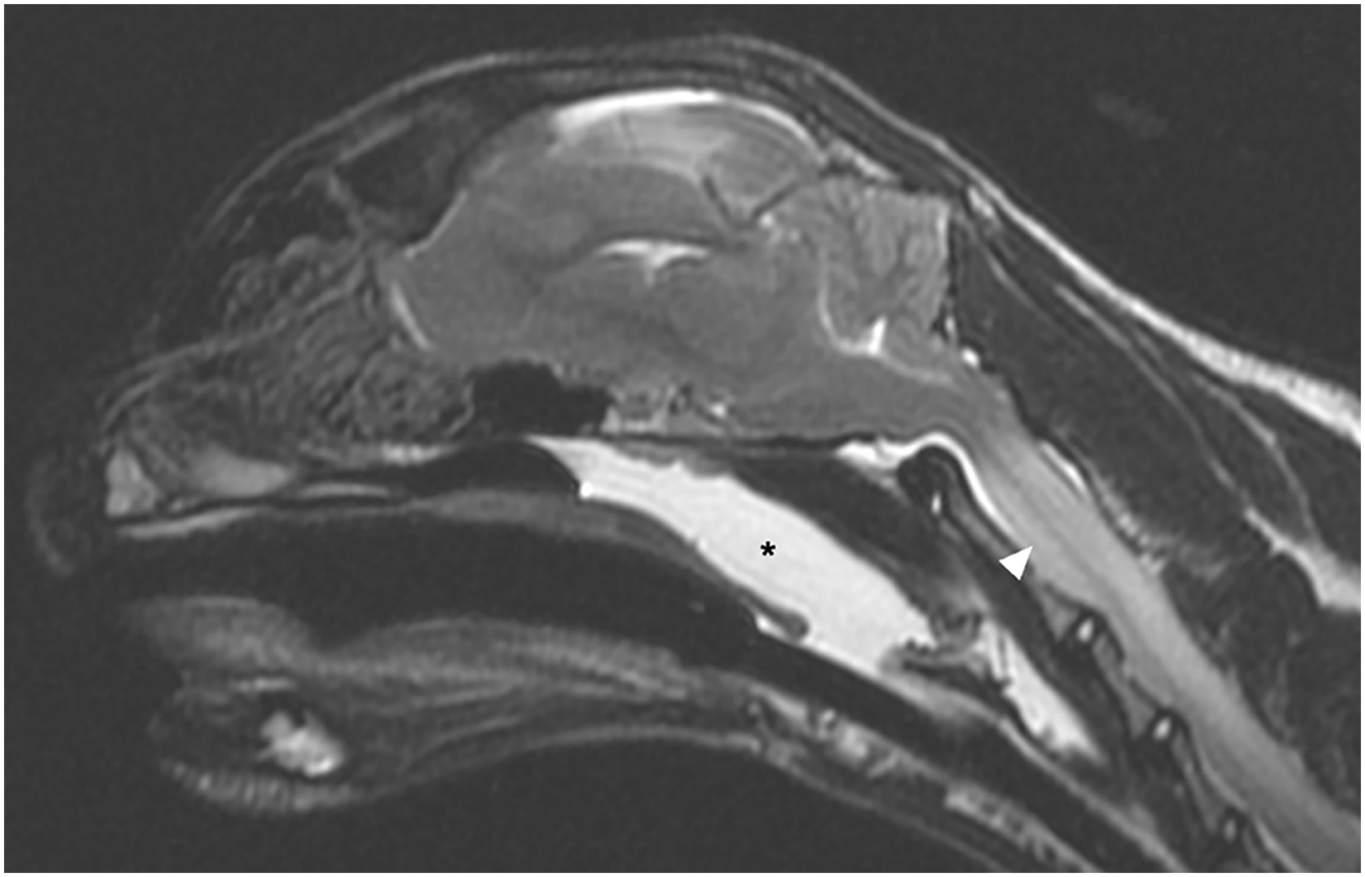
Mid-sagittal T2-weighted (T2W) image of the brain. Note the mass effect with subfalcine, as well as caudal transtentorial, and transforaminal herniation. There is a severe cervical spinal cord swelling and parenchymal T2W hyperintensity (arrowhead). The nasopharynx is fluid filled (*)

**Figure 4:**
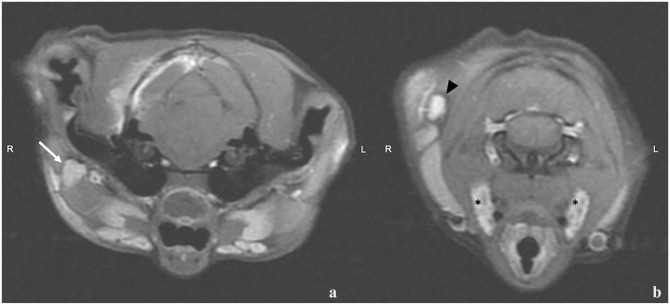
Transverse T1-weighted postcontrast images of the brain at the level of (a) the tympanic bulla and (b) the atlantooccipital junction. Note the moderately enlarged right parotid (arrow), right lateral retropharyngeal (arrowhead) and bilateral medial retropharyngeal (*) lymph nodes

An MRI examination revealed a right-sided, diffuse swelling of the cerebral grey matter that formed a focal, ill-defined mass lesion at the level of the right piriform lobe (intra-axial) (Figures 1 and 2a). The lesion was iso- to mildly hyperintense in the fluid sensitive sequences and T1W iso- to mildly hypointense compared with the cerebral grey matter. The mass was partially surrounded by an irregularly shaped and focally discontinuous, thick T2W and FLAIR hyperintensity that affected the pachy- and leptomeninges (extra-axial). A dural tail sign with leptomeningeal enhancement and widening of the sulci could be observed after contrast administration (Figure 2b). There was mild perilesional and moderate distant hyperintensity in the fluid sensitive sequenced without signs of restricted diffusion in the region of the white matter, consistent with a vasogenic oedema. The lesion caused a marked mass effect with left-sided shift of the cerebral falx (subfalcine herniation) and compression of the right lateral, third and fourth ventricle. The thalamus, brainstem and the cerebellum were caudally displaced and compressed with resulting caudal transtentorial forebrain herniation and transforaminal herniation of the cerebellum (Figure 3). The cervical spinal cord was diffusely swollen and had a parenchymal T2W hyperintensity and T1W hypointense central cavity extending from C2 to C5, consistent with a syringomyelia and spinal cord oedema, most likely secondary to flow disturbances of the cerebrospinal fluid (CSF) at the level of the mesencephalic aqueduct and foramen magnum. At the level of the right frontotemporal lobe, the calvarium was severely irregular defined and osteolytic. The right temporal muscle was moderately swollen and markedly contrast enhancing. The unstructured hyperintensity along the calvarium was indistinguishable from the above muscular lesion (Figure 2). The right parotid, right lateral retropharyngeal and both medial retropharyngeal lymph nodes were moderately enlarged and had strong contrast enhancement (Figure 4).

The imaging summary included an intra-axial right-sided space-occupying lesion at the level of the piriform lobe with diffuse regional and distant extra-axial infiltration, mild perilesional- and moderate distant vasogenic oedema. In addition, there were aggressive calvarial osteolysis, and infiltration of the frontotemporal bone as well as of the right temporal muscle, and finally, enlarged, contrast-enhancing regional lymph nodes. Based on these imaging findings, main differentials included neoplasia such as histiocytic sarcoma (HS) or T-cell lymphoma. Aggressive meningioma or glioma had to be considered. Owing to the involvement of bone and muscle, a primary bone tumour, such as osteochondrosarcoma or other sarcoma, was included. Other neoplastic diseases, such as granular cell tumour or meningeal carcinomatosis, were considered less likely. Because of the geographical location (Switzerland) and no travel history, a fungal disease was considered unlikely. The enlarged lymph nodes most likely represented metastases of any of the above suggested neoplastic processes, as non-neoplastic diseases such as granuloma, abscess formation secondary to fungal or bacterial infection and thus reactive lymphadenopathy appeared less likely.

**Figure 5:**
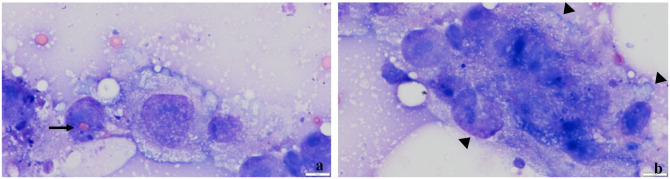
Fine-needle aspiration smears of the right temporal muscle (a,b). There is a moderate amount of histiocytes with a moderate amount of pale, blue and partly finely vacuolated cytoplasm. (a) Their nucleus is oval and has a coarse chromatin structure. Note the phagocytised erytrocytes and precursor cells of the erythropoietic cell series (arrow). There is moderate anisocytosis, anisokaryosis and anisonucleoliosis. Occasionally, multinucleated giant cells are present (b) (arrowheads). Modified Wright’s stain, bar = 10 µm

Ultrasound-guided fine-needle aspirations of the right temporal muscle and the right parotid lymph node were performed. The smears were dried and stained with modified Wright’s stain. Cytology revealed in both the temporal muscle and the right parotid lymph node a moderate amount of round to oval cells with a moderate amount of pale, blue and partly finely vacuolated cytoplasm with an oval nucleus with a coarse chromatin pattern (Figure 5). Occasionally, phagocytised erythrocytes and precursor cells of the erythropoietic cell series were found within these cells (Figure 5a). The latter were further characterised by moderate anisocytosis, anisokaryosis, as well as occasional megalocytes (Figure 5b). Based on the morphology of the cells, the cytological diagnosis was HS with metastasis into the right parotid lymph node.

Given the clinical condition of the patient, the extensive MRI findings and the poor prognosis, the owners decided to euthanase the cat. A post-mortem examination was declined by the owner and not performed.

## Discussion

HS is a malignant neoplasm that develops from proliferation of malignant histiocytes originating from myeloid dendritic antigen-presenting cells or macrophages.^1–3^ HS can occur either localised or disseminated.^1,4^ The localised form originates from a single site, most commonly in the soft tissues along the limbs, and shows a locally invasive growth usually with metastasis to the regional lymph nodes.^5^ The disseminated form is a multisystemic disease that is most often found in the spleen, liver, lung, bone marrow, lymph nodes and other organ systems, including the central nervous system (CNS).^4,5^

Feline HS involving the CNS is a rare disease described in only a few case reports to date.^6–10^ MRI features were described as either diffuse meningeal involvement with contrast enhancement or as isolated extra-axial mass lesion formation.^6,9,10^ Intracranial masses were described to be well- to ill-defined, T1W hypo- and T2W hyperintense, strongly contrast enhancing with dural tail sign and mild perilesional oedema formation.^6,10^

The intracranial component of the present case had similar MRI features as previously described in dogs and in few case reports of cats.^3,4,9–13^ There was a diffuse leptomeningeal extension and contrast enhancement with concurrent widening of the sulci and an additional focal, ill-defined, non-enhancing mass in the right piriform lobe, as well as perilesional and distant oedema formation.

In this case, the extensive focal calvarial osteolysis with associated muscular involvement represents a novel feature that has not been previously reported in association with intracranial HS, in neither cats nor dogs. It is well known that HS of the axial and appendicular skeleton has an aggressive biological behaviour with soft tissue mass formation, associated bone destruction and regional lymph node metastasis.^14^ The pathophysiology of tumour-associated osteolysis is not exactly understood; however, three mechanisms are suggested: (1) direct tumour invasion; (2) tumour associated bone resorption caused by proteolytic enzymes (ie, matrix metalloproteinases); and (3) pressure atrophy. In the present case, it is assumed that direct tumour invasion was responsible for the calvarial osteolysis. Up to now, intracranial tumour expansion outside the calvarium through a skull defect has been rarely reported in veterinary literature and only in association with meningiomas and gliomas.^15–19^

In the cat presented in this case report, the muscular lesion had similar signal intensities when compared with the diffuse meningeal lesion and were almost indistinct or non-separable from it. This finding could suggest a primary muscular neoplasm with infiltration of the bone and meninges, possibly mirroring an aggressive biological behaviour as mentioned above. However, as the MRI characteristics of the mass lesion at the level of the right piriform lobe were, apart from the lack of contrast enhancement, almost identical to the features described in CNS HS in dogs and cats, a primary CNS tumour with secondary osteolysis and muscular invasion was considered the most likely differential diagnosis.^3,4,9–13^ Unfortunately, a post-mortem histopathological evaluation of the brain was not performed; therefore, it remains open whether a necropsy could clarify if the brain mass was primary or secondary in nature.

Regardless of the origin of the primary tumour, if muscular or intra-/extra-axial, the cat presented in this case report most likely had a local form of HS as, to the best of our knowledge, the extensive lesion at the level of the right piriform lobe with local invasion and metastases into the regional lymph nodes was the only one identified.

The diagnosis of HS was confirmed by cytology of the muscular lesion and the parotid lymph nodes. A limiting factor of the investigation of this case was the lack of pathological examination with histopathology, which is currently used as a standard technique for HS. However, intraoperative cytology using the smear technique is described to provide an accurate, fast diagnosis for nervous system tumors.^20^ In particular, five CNS HSs in four dogs and one cat were investigated with this technique and considered diagnostic.^20^ Therefore, cytologic investigation could be considered to be an interesting alternative to histopathology. The diagnosis could even be confirmed by using immunocytochemistry; unfortunately, in our case, there were not enough stained smears available to perform this technique. But based on the larger amount of pale blue cytoplasm and the rather coarse chromatin pattern, these cells were interpreted as histiocytic rather than lymphocytes. Lymphocytes tend to have a fine chromatin pattern, normally round to slightly indented nuclei, with only a small rim of dark blue cytoplasm.

## Conclusions

This case report adds novel MRI features of HS involving the CNS in cats with extensive aggressive calvarial osteolysis and regional muscular invasion, which were not previously described. The intracranial component of the HS in our cat shared similar MRI features as those published in the veterinary literature to date. A diagnosis was achieved via cytology and cytology should be taken into account in such cases. HS should be considered as a differential diagnosis for an intra-axial mass with additional diffuse meningeal infiltration (extra-axial), regional aggressive bone lysis, adjacent muscular invasion and regional lymphadenopathy.
